# Correction: Tabouret et al. TP5, a Peptide Inhibitor of Aberrant and Hyperactive CDK5/p25: A Novel Therapeutic Approach against Glioblastoma. *Cancers* 2020, *12*, 1935

**DOI:** 10.3390/cancers17193094

**Published:** 2025-09-23

**Authors:** Emeline Tabouret, Herui Wang, Niranjana Amin, Jinkyu Jung, Romain Appay, Jing Cui, Qi Song, Antonio Cardone, Deric M. Park, Mark R. Gilbert, Harish Pant, Zhengping Zhuang

**Affiliations:** 1 Neuro-Oncology Branch, Center for Cancer Research, National Cancer Institute, National Institutes of Health, Bethesda, MD 20892, USA; herui.wang@nih.gov (H.W.); jinkyu.jung@nih.gov (J.J.); jing.cui@nih.gov (J.C.); qisong@fudan.edu.cn (Q.S.); mark.gilbert@nih.gov (M.R.G.); 2 Team 8 GlioME, CNRS, INP, Inst Neurophysiopathol, Aix-Marseille University, 13005 Marseille, France; romain.appay@ap-hm.fr; 3 Neuronal Cytoskeletal Protein Regulation Section, National Institute of Neurological Disorders and Stroke, Bethesda, MD 20824, USA; aminn@ninds.nih.gov (N.A.); panth@ninds.nih.gov (H.P.); 4 University of Maryland Institute for Advanced Computer Studies, College Park, MD 20742, USA; antonio.cardone@nih.gov; 5 Department of Neurology and the Committee on Clinical Pharmacology and Pharmacogenomics, The University of Chicago, Chicago, IL 60637, USA; dpark9@neurology.bsd.uchicago.edu

In the original publication [1], there was a mistake in Figure 2B-LN229 as published. An unintentional error occurred during the cropping of the six-well plate images used to display individual wells for the LN229-1 µM and 10 µM conditions. The corrected Figure 2B-LN229 appears below. The authors apologize for any inconvenience caused and state that the scientific conclusions are unaffected. This correction was approved by the Academic Editor. The original publication has also been updated.

## Figures and Tables

**Figure 2:**
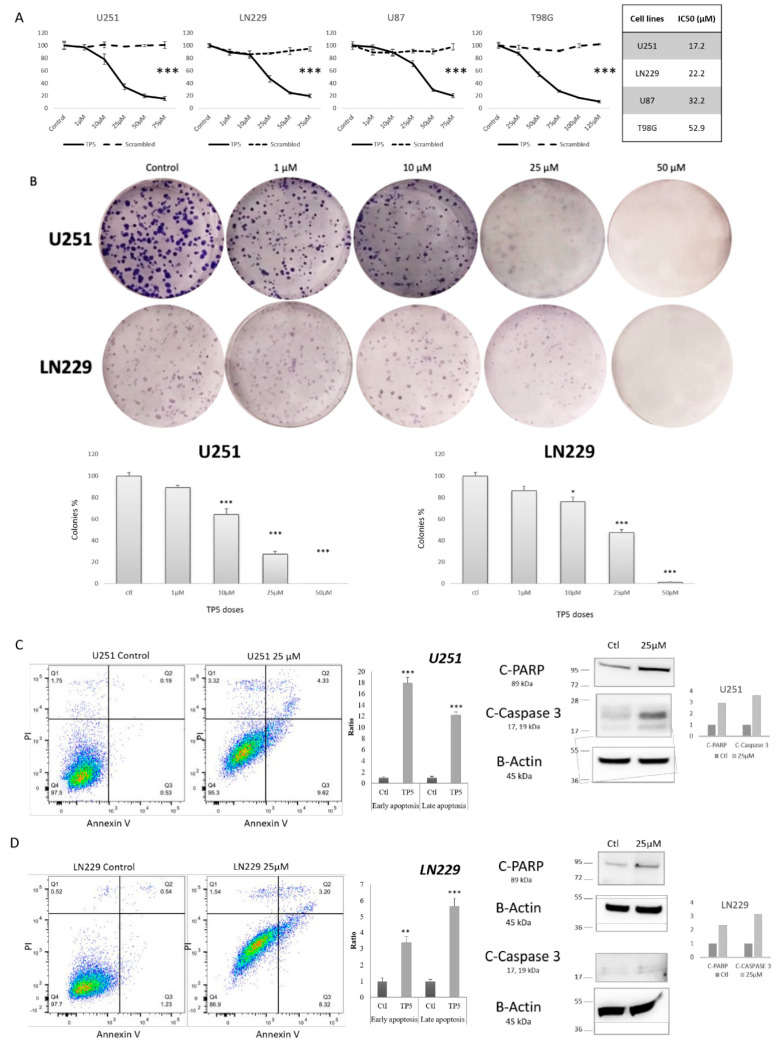
TP5 decreases cell viability by inducing cell apoptosis. (**A**) Viability of U251, LN229, U87 and T98G cells treated by indicated concentrations of TP5 or scrambled peptide for 72 h is shown (*** *p* > 0.001). Right table: IC50 concentrations. (**B**) Clonogenic growth of U251 (top panels) and LN229 (bottom panels) cells is shown. The bar graphs display quantification of colonies under treatment at indicated concentrations (*N* = 3) (* *p* < 0.05; *** *p* < 0.001). (**C**) Apoptosis analysis by Annexin V/Propidium Iodide (PI) staining. Left panel: Representative flow cytometry dot plot graphs of annexin V and PI in U251 cells are shown. The bar graph displays the quantification of early (Q3) and late (Q2) apoptotic cells after treatment by TP5 (25 µM) in U251 cells (*N* = 4) (*** *p* < 0.001). Right panel shows protein level of cleaved PARP and cleaved Caspase 3 in U251 cells after 24 h of treatment by TP5 (25 µM). (**D**) Apoptosis analysis in LN229 cells. Left panel: Representative flow cytometry dot plot graphs of annexin V and PI in LN229 cells are shown. The bar graph displays the quantification of early and late apoptotic cells after treatment by TP5 (25 µM) in LN229 cells (*N* = 4; ** *p* < 0.01; *** *p* < 0.001). Right panel shows protein level of cleaved PARP and cleaved Caspase 3 in LN229 cells after 24 h of treatment by TP5 (25 µM).
